# What Is Going on with Visual Attention in Reading and Dyslexia? A Critical Review of Recent Studies

**DOI:** 10.3390/brainsci12010087

**Published:** 2022-01-10

**Authors:** Conrad Perry, Heidi Long

**Affiliations:** Faculty of Health and Medical Sciences, School of Psychology, The University of Adelaide, Adelaide 5005, Australia; Heidi.d.long@gmail.com

**Keywords:** vision, attention, reading, dyslexia, critical review

## Abstract

This critical review examined current issues to do with the role of visual attention in reading. To do this, we searched for and reviewed 18 recent articles, including all that were found after 2019 and used a Latin alphabet. Inspection of these articles showed that the Visual Attention Span task was run a number of times in well-controlled studies and was typically a small but significant predictor of reading ability, even after potential covariation with phonological effects were accounted for. A number of other types of tasks were used to examine different aspects of visual attention, with differences between dyslexic readers and controls typically found. However, most of these studies did not adequately control for phonological effects, and of those that did, only very weak and non-significant results were found. Furthermore, in the smaller studies, separate within-group correlations between the tasks and reading performance were generally not provided, making causal effects of the manipulations difficult to ascertain. Overall, it seems reasonable to suggest that understanding how and why different types of visual tasks affect particular aspects of reading performance is an important area for future research.

## 1. Introduction

Visual attention is an important part of reading and deficits related to it have been commonly implicated in dyslexia [1,2,3]. In this critical review, we wanted to investigate the current issues in this area. The main goal was to examine the extent to which researchers have converged on particular types of experiments and experimental designs that can be used to examine visual attention and how it interacts with specific processes involved in reading. This is important because numerous tasks have been historically used. Finding those that have high validity and can be meaningfully used to examine different aspects of the reading system in different populations would be very useful. We attempted to go some way towards this by evaluating recently published studies and their results.

### What Is Strong Evidence That Would Show Visual Tasks Predict Reading Performance?

We first need to consider the type of experimental design required to examine the extent to which visual processing is important in reading when compared with other factors. There are a number of criteria that one might want. Notably, it is very well established that different types of deficits in reading are significantly correlated [4], even if they might seem conceptually separate (e.g., visual and phonological deficits). This includes data from large samples [4,5]. These correlations are important because without control variables being run concurrently, it makes the possible causal component of some tasks difficult to ascertain (e.g., the visual attention part of a visual attention task). Perry et al. [6] showed how serious this problem was using a computational model of reading. They showed that a very similar pattern of mean error rates from dyslexic and normal readers could be obtained with the model using two entirely different parameters to simulate the deficits—one related to phonology and the other related to vision. Thus, without having information about both deficits, it would not be possible to accurately determine the effect size of either.

Given that deficits tend to be correlated, a gold standard would be to identify the amount of variability that a visual deficit could capture over and above commonly accepted measures used to examine reading performance believed to be important but not directly related to visual processing. The most reasonable of these are results from phonological awareness (PA) and rapid naming (RAN) tasks. Whilst standard PA tasks are well understood and extensively used [7], factors loading on the RAN task are less clear, although there are good hypotheses that suggest phonology is important [8,9]. Thus, the RAN task is suitable for finding children who have phonological problems that cause them to be both slow at reading and language tasks in general. However, other types of deficits, including visual attention deficits, could also potentially cause children to read slowly. Thus, if a slow reader is found, using a RAN task allows a better understanding of why this is the case. In particular, if a child shows normal performance on a RAN task but poor performance on a visual task, then, with other supporting evidence, one might conclude that they are a slow reader because of a visual deficit. Alternatively, if they are slow on a RAN task but also have a visual deficit, one would need further evidence to work out the extent to which a phonological deficit, a visual deficit, or both are responsible for their slow reading. Thus, using RAN can help distinguish between those who are slow readers and have specific visual processing problems and those who are slow due to other problems.

A worthwhile but far less commonly used design to examine reading deficits is to compare dyslexics to both reading age (RA) and chronological age (CA) controls. This may provide insights into reading that may be less obvious than when only a chronological age control group is used. In the dyslexia literature, such a design was historically used to try and understand how different types of dyslexia may arise [10,11], while it has also been used to examine properties of exogenous attention [12].

Apart from which groups should be compared, when qualitatively different groups are used (e.g., dyslexics vs. controls) it is also useful to see the correlations with the groups both separately and together. This can provide useful information about whether a particular effect might be nonlinear. For example, some form of processing may reach a level in normal readers where essentially all are at ceiling. That type of processing would thus show low predictive power on reading measures. However, it may still be at a level which causes differences with dyslexics and younger readers. Similarly, there may be qualitative differences between dyslexics that would be rarely seen in normal readers. This would again be hard to see without separate analyses being conducted on the groups. Finally, if mean differences are found between groups but there are no significant correlations within groups, it may simply indicate that one group tends to be competent at most tasks and the other group less competent at most tasks.

## 2. Papers Used in Our Review

To examine recent studies on visual attention and the extent to which they fulfilled our criteria described above, we searched for recent papers (2019–2021). We also added a number available to us as preprints or that we knew existed but were missed in the searches and were relevant to the discussion, plus a small number from 2018 that were very useful for comparative purposes with other studies found in the search. We did not add every paper from 2018 that would have been found in a search that included them. This was because the initial search results suggested that the papers we used were sufficient to meaningfully evaluate the themes that had emerged from them without proliferating ideas that occurred more rarely and would need more investigation to evaluate thoroughly. We used the following queries to search for articles in mid-April 2021:

Search 1

PUBMED search.

Keywords; dyslexia-visual-attention.

The first 50 relevant papers published after 2019.

Search 2

We used a Google Scholar search limited to 2021 with all papers that mentioned dyslexia in their abstract and the following keywords:

(a) Visual—attention span (searched first 200 papers, until there were no more relevant papers appearing);
(b) Dyslexia—statistical learning (no more relevant papers appearing after 130 papers);
(c) Developmental—dyslexia—attention (no more relevant papers appearing after 110 papers);
(d) Dyslexia—visual attention—deficit (no more relevant papers appearing after 220 papers);
(e) Dyslexia—visual attention—processing (no more relevant papers appearing after 160 papers).

From this list, we removed papers if:

(a) They were not obviously related to visual processing or visual attention;
(b) They involved reading in non-Latin alphabets;
(c) The participants had brain damage;
(d) The paper was not available in English;
(e) The central factor of investigation was schizophrenia, autism, or perceptual or visual impairment;
(f) They were published in a non-peer-reviewed journal;
(g) They had no publisher listed.

The papers we reviewed in the main text are marked with an ‘*’ in the References. To keep the review focused, we also placed descriptions of papers that we reviewed related to ocular movements and remediation studies, plus a small amount of relevant commentary, into Supplementary Materials (Listing S1). This was because papers in these areas typically provided less insight into potential causal routes between visual processing and particular aspects of reading than the other papers. Their references also occur away from the main text in Supplementary Materials. The main reason we did not review papers with languages that used non-Latin alphabets was because they may have different requirements in relation to visual attention, especially because most of the papers in this category featured reading in Chinese. Papers using Cyrillic and Greek alphabets would have been largely comparable to Latin alphabets, but these did not turn up in our search. Whilst we did find a number of papers using Devanagari, Devanagari may be less comparable to Latin alphabets [13].

## 3. Research Articles

The most commonly used task from our literature search was the Visual Attention Span (VAS) task using alphanumeric digits. This task was initially used to measure the number of contiguous letters that can be processed in parallel [3], although non-letter and non-linguistic stimuli have also been examined in a similar way. VAS has also been examined by Lobier et al. [14] in conjunction with the Theory of Visual Attention [15], which separately measures visual processing speed and visual short-term memory capacity. Lobier et al. showed that the VAS task could explain all of the variance associated with reading speed that the TVA task could, and that part of the VAS task’s performance could be explained by visual short-term memory capacity. However, visual short-term memory capacity by itself was not a significant predictor of reading speed once results from the VAS task were accounted for. At least in reading research, the VAS task might thus be considered a good alternative to the more complicated TVA task. Unsurprisingly, our search also found other types of tasks that were used. These included:

(a) Inhibition of return (IOR) tasks, which have been used to examine the ability of people to orient their exogenous attention [2,12,16,17]. They measure reaction times of stimuli that follow a cue that causes a proceeding stimulus to appear in an expected or unexpected spatial position. A no cue or neutral cue condition may also be used. This type of task allows both benefit-related and cost-related effects of exogenous attention to be examined. When short cue durations are used, typically people respond fastest when the target is in the expected position based on the cue, unlike when long cue durations are used, where there may be a reduced or no effect. This suggests their exogenous attention has been quickly engaged and allows them to benefit from a correct cue. In cases where the cue is in the incorrect position, people typically respond more slowly. This suggests they need to reorient their attention to the correct spatial position when the target appears. If someone shows little effect of a short-duration cue but a difference with a long-duration cue, or a comparatively reduced cueing effect compared to other people, it is referred to as sluggish attention and suggests that they are slow to orient their attention [12,16]. In terms of reading, it has been suggested that this type of task measures the ability of people to orient their attention onto different parts of letter strings from which graphemes can then be extracted, which is especially important in pseudoword reading [2].
(b) Attentional blink (AB) tasks, which are meant to be a measure of temporal sequencing and temporal attention [18].
(c) Visual search tasks. It has been suggested that processes used in these tasks are related to the underlying skills needed for the left-to-right scanning of text and the extraction of graphemes from letters [19].

## 4. Results from the Studies

### 4.1. Studies including the VAS Task

One of the larger studies examining visual attention that met our gold standard of using a PA and RAN task as controls was by Cirino et al. [20]. They looked at 90 students who were at a high risk of having a reading disorder (mean age: 13.7 years) using letter, number, and Hiragana stimuli with a VAS, visual search, attentional blink, and an IOR task. They then compared them to single-word reading and reading fluency scores. The results from the VAS task with letter targets were significantly correlated with single-word reading, explaining around 6% of the variance after RAN, PA, and age were controlled for, but no significant effect was found with sentence fluency. With number stimuli, significant effects were found in both the single-word reading (7% of the variance) and the fluency task (4% of the variance). With Hiragana stimuli, no significant effects were found. The visual search task was the only other task that was a significant predictor of reading scores after PA and RAN were controlled for. With sentence fluency, it was significant when letters (3% of the variance) and numbers (5% of the variance) were used as targets. Alternatively, no significant effects were found with any type of single-word reading or when Hiragana stimuli were used.

Interestingly, the correlations between the visual tasks Cirino et al. [20] used were weak, despite all having been claimed to measure some form of scanning or temporal sequencing. Notably, of the eight different variables examined in the attentional blink task, none correlated significantly even at the uncorrected level with the three visual search tasks. This is somewhat surprising because when Ruffino et al. [21] examined visual-spatial attention using an IOR task and temporal attention with a relatively similar task, they found results from both types of attention were correlated (*r* = −0.295). Relationships between the attentional blink and IOR task were slightly stronger but still rather weak, where, of the 60 possible comparisons, there were three that were significant at the uncorrected level and one (*r* = 0.46) that would have been significant even after Bonferroni correction. There was also only one significant correlation at the uncorrected level found between results from the visual search task and the IOR task (*r* = 0.23). This is somewhat surprising because the visual search and the IOR task are related, as both use visual-spatial attention. Notably, Cirino et al. set up their IOR task so that it manipulated exogenous attention and their visual search task used number, letter, and Hiragana stimuli. At least for the number and letter stimuli [22], which would have been very well learnt by their participants compared to Hiragana, performing the visual search task should thus have also used exogenous attention to some extent. It is therefore surprising that no significant correlations between these two tasks were found. Given the lack of correlations across tasks, this suggests either (a) the tasks are unreliable, which seems very unlikely given their use in other areas; (b) they are measuring entities that are more different than one might infer from their descriptions and use in the reading literature; or (c) with a normal population, the differences between them are so small that correlating their scores only produces weak results. This also seems unlikely given their historical usage. Furthering our understanding of how these tasks differ and how these differences might be related to processes involved in reading would thus be very useful.

Some studies have also tried to look at causal effects of visual attention on reading by using longitudinal designs. Notably, van den Boer and de Jong [23] ran a longitudinal study in Dutch looking at VAS scores and how they related to word and text reading in Grade 4 children (mean age: 8 years 11 months), using PA and RAN as controls. They found that VAS explained a relatively small amount of variance with word (5.9%), pseudoword (6.3%), and text reading (5.9%), and that the stability of the task was high across their two time points approximately one year apart. After further analyses, van den Boer and de Jong claimed that VAS and reading development were ‘largely independent’ (p. 439). They suggested that VAS might be a correlate of rather than a predictor of reading performance, at least for their demographic. Valdois et al. [24], alternatively, claimed VAS was predictive of future learning. They looked at whether scores from kindergarten children (mean age: 5 years 10 months) predicted reading fluency in Grade 2 (approximately one year later). Their results showed that when looking at the effect of letter name and sound knowledge, PA, and VAS as a group, the three factors accounted for 6.3% and 4.6% of the variance in pseudoword and irregular word reading fluency, respectively, over and above early literacy knowledge. When looking at PA and VAS as a group over and above early literacy knowledge and letter naming, they found PA and VAS explained 4.7% of the variance in text reading fluency. In addition, in the different groups of variables, VAS was clearly the strongest predictor, although the significance of individual predictors was not given and nor was RAN used as a control. These results are interesting when compared to van den Boer and de Jong’s because Valdois et al. suggested they meant that causation could be found with younger children.

Another reason visual attention tests may be useful is if they can predict not just learning effects but deficits in disordered groups. Valdois et al. [5] examined this with French children using a sample of 279 French 6th-graders. They found that VAS explained 6%, 8%, and 6% of the variance above RAN, PA, and other controls in single word, pseudoword, and text reading, respectively, whereas RAN and PA together contributed 20%, 31%, and 20% on the same tasks. Importantly, they also looked at the prevalence of deficits in those who were poor readers and found they occurred 18%, 20%, and 15.5% of the time with the VAS, PA, and RAN tasks, respectively. There were also intercorrelations between the deficits that occurred at a rate higher than would be predicted by chance, suggesting that multiple deficits were common. At least for the proportion of times the individual deficits occurred, these numbers are somewhat different to studies examining the amount of variance these factors account for, which tend to find VAS accounts for less variance than PA and RAN.

Using Dutch 10th-graders (mean age: 16.5 years) who had an early or late diagnosis of dyslexia and PA, RAN, and VAS tasks, Bazen et al. [25] also examined the extent to which dyslexics had particular disorders. They found 31% of their sample had a VAS weakness and 63% and 60% had RAN and PA weaknesses, respectively. This is similar to the pattern found in terms of the amount of variance that is accounted for by these factors in other studies (i.e., RAN and PA account for more than VAS), although it differs slightly to the deficit results found by Valdois et al. [5] in French.

VAS has also been examined in a number of studies where the statistics were somewhat less comparable. One of the larger ones was by van der Kleij et al. [26], who examined Dutch children (mean age: 10.5 years) after they had been treated for dyslexia. They found that there were significant zero-order correlations of VAS with word-reading accuracy (*r* = 0.26) and pseudoword-reading accuracy (*r* = 0.35), which is similar to the size of zero-order correlations in some other studies, but fluency scores for the same variables were not significant (*r* = 0.19 and *r* = 0.21, respectively).

Overall, at least for the studies we reviewed, most found small but significant effects of VAS on at least some reading measures. This was despite the large age range (kindergarten to adults) and different languages used. However, the studies did not typically consist of very severe dyslexics where VAS effects may be weaker in French [27], nor were they conducted in German where a recent study examining dyslexics versus controls did not find any significant differences [28]. The similarity across the age range is slightly surprising. This is because one might have suspected that the distribution of those who remain dyslexic as they age would differ from those who are dyslexic as young children. In this case, older dyslexics are likely to have had some form of remediation, yet still have reading difficulties. Alternatively, some young children with reading difficulties who receive training to improve their reading would presumably benefit from it, thus removing them from the older dyslexic pool. This means that some more moderate cases of dyslexia may be removed from older pools leaving those with more serious problems. This could change the sampling distribution of the pools, yet this was not obvious in the data. Alternatively, it is possible that some of the younger dyslexics in the samples may not have had any remediation, in which case they may show more dysfunction than even dyslexic adults who had some remediation but did not receive enough help to reach a normal reading level.

A number of further observations can be made from the VAS task. One is that the theory behind the task was based on a model of reading [29] that has not been extended or evaluated as well as other models, although many of the papers used terminology from those models and presumably this in part reflects the way people think about reading. Further work on how the paradigm might be understood from the point of view of the Triangle model [30], the Connectionist Dual-Process model [31], and the Dual-Route Cascaded model [32] may thus be useful. Cirino et al. [20] also noted that the VAS task is affected by more variables than simply letter span, and that it is likely to pick up at least some effects of phonology. The extent to which the VAS task measures processes above and beyond just letter span would thus be useful to know. Whilst we did not intend to review papers in Chinese, a recent example where VAS is very likely to be predicting more than just character span comes from Huang et al. [33]. They found it predicted single-character reading fluency for a group of relatively young readers (mean age: 8.4 years). This means if VAS is taken as a ‘window’ in Chinese, children in the youngest group must have a window less than one character some of the time, otherwise a correlation could not be found. Since this seems rather unlikely given their age, it suggests there must be further processes associated with the VAS task not related to just character span.

One way of examining what the VAS task measures is by using cross-language comparisons with tasks controlled across languages (e.g., Awadh et al. [34]), such as occurred with the pioneering studies looking at orthographic depth where multiple languages were compared [35]. In this case, properties such as the maximum-sized window needed for processing to allow for the extraction of graphemes in pseudowords could be examined. The maximum size this window needs to be differs across languages and may need to be longer than the longest grapheme because the behaviour of some graphemes in some languages is contextually sensitive based on the other letters around them. Thus, the only way they can be converted into phonology reasonably is to take this context into account. For example, in Australian English, the -ed in -oiled always causes two syllables (e.g., *boiled*) but the -ed in -eeled does not (e.g., *wheeled*). Thus, even though the largest grapheme in Australian English is three letters long (e.g., -eer), a window larger than three letters is needed. In particular, in terms of the maximum window size needed, in English it is five [36], in Italian it is three [37], and in Dutch it is four. Given such differences between languages, one could examine the effect of window size on word and pseudoword processing. With Italian, one might predict pseudoword processing would be less affected by VAS than languages like English given the smaller window needed. Within language comparisons could also be informative. In particular, the effect of VAS in English with the same type of task but different stimuli difficulties would be interesting. This is because it has been shown that the size of the units that people use when reading differs depending on the list context [38]. When irregular words are used in a list with pseudowords, people tend to use larger groups of letters with the pseudowords than when they are not, which suggests that the size of the window and indeed what is extracted from them may differ. The extent to which VAS performance predicts these could potentially give evidence for different-sized windows, different efficiency of processing, or different flexibility in window use. For example, if the widest-sized window is not needed to read some English stimuli sets because easy stimuli are used, it would be interesting to know if people became more efficient at processing. Similarly, if very difficult stimuli which are likely to cause people to default to simple spelling—sound mappings are used [39], it would be interesting to know the extent to which this affects words that are not so difficult to process. In this case, the window size might be smaller than would otherwise be optimal.

### 4.2. IOR/Conflict Tasks

Franceschini et al. [16] examined three different tasks with Italian adult dyslexics. Two of their tasks have been claimed to measure different aspects of the reading system which are likely to differ between dyslexics and controls: an IOR task using word/pseudoword stimuli as targets where lexical decision was performed on them and a coherent dot motion task which they suggested is related to the encoding of letter position. A third luminance grating task was also used as a control because they expected to find no differences between the performance of dyslexic and normal readers using it. Their results showed the IOR and coherent motion task produced fairly weak differences or null effects between the dyslexic and control groups. Differences such as those found in the right visual field, which have been reported with dyslexics by others in IOR tasks [2], were not found, although an overall left-hemisphere advantage was found. Less surprising was that the luminance grating task did not produce differences. They claimed their results showed that Italian readers could compensate for visual attention deficits that may be due to the early processing of letters. In particular, they suggested they could achieve this by becoming very efficient at lexical (i.e., whole-word) access from orthography. Thus, even if they do have problems processing at the letter level, downstream processes may become so efficient such that these difficulties do not cause markedly slower reading or higher error rates than normal. This set of tasks would be interesting to run with English readers. This is because English readers lexicalise words much more slowly than Italian readers due to the large orthographic depth difference between the two languages, leading to poorer performance on both word and pseudoword reading [35]. Given an otherwise identical replication in English, presumably one would expect the results would not show such weak effects, as English speakers would have greater difficulty compensating for the weakness. Another change to the experiment that may be worthwhile would be to use a naming rather than a lexical decision task. In this case, pseudoword latencies would be of greater interest than when a lexical decision task is used given a naming task forces people to generate phonology. This is important given previous work using this type of task has suggested that focused visuo-spatial attention has a special role in pseudoword reading [2].

Rima et al. [40] were also interested in the effect of congruent and incongruent stimuli, as well as the hemifield the stimuli were processed in. To examine this, they manipulated temporal frequency using drifting Gabor patches in both hemifields with normal and dyslexic readers, causing attention to be allocated from left to right or right to left. Their results indicated that both normal readers and dyslexics showed an advantage in the right visual field when attention was manipulated from left to right, but dyslexics showed a smaller advantage. No advantage was found in either group when attention was manipulated from right to left. The authors claim these results are likely to be caused by perceptual learning rather than visual attention.

### 4.3. Other Tasks

Of the experiments which did not explicitly use a VAS or an IOR task, the one by Stefanac et al. [41] was most similar to a task measuring VAS. Their study was also the only one that used dyslexics (8–16 years, median age: 12.3 years) with both reading-matched and age-matched controls. They tested their sample using a task from the Theory of Visual Attention [15], which estimates visual processing speed and capacity measures. In terms of the specific comparisons between dyslexic and control groups, no overall effect of visual working memory capacity was found compared to either CA or RA controls. Alternatively, the dyslexics’ processing speed was found to be slower than CA but not RA controls, which they suggested meant that development dyslexics had a domain general processing speed deficit. Comparing such a slowdown with a RAN task would have been useful both for theoretical and for practical reasons—the TVA task is slow to run, and thus if the processing speed component is similar to the RAN or the VAS task, it would obviate the need to use it in studies with younger children where such time constraints are often important.

Stefanac et al. [41] also used a design that was uncommon in the studies we examined, combining results from different types of words to form a new dependent variable. This gave them an idea of lexical profiles, which is theoretically justifiable based on all major reading models (note that Valdois et al. [5] also used lexical profiles calculated in quite a different way, but found they had no significant effect). To achieve this, they measured the error rate of regular words, irregular words, and pseudowords, a distinction of historical importance [10,42]. They then created a measure by taking the correct counts from pseudowords, subtracting the correct counts from irregular words, and then dividing that number by the total number of words used. The idea behind this is that irregular words tap lexical usage and pseudowords tap sublexical usage (i.e., GPC rules or sublexical spelling–sound associations). Thus, if someone is good at reading irregular words compared to pseudowords, they have a lexical dependency. They showed that the speed of processing differences were largely caused by participants with poorer lexical skills (i.e., ability to read irregular compared to nonwords) and, importantly, that these differences were not restricted to linguistic stimuli. Using data from both control groups then allowed them to suggest that the reason for this may be the inability of dyslexics to refine their use of attention early in reading development.

Apart from visual-spatial attention, Provazza et al. [43] specifically examined visual working memory in English adult dyslexics. They found their sample of dyslexics were impaired on two of three visual working memory tasks, one of which involved static stimuli and the other which involved sequentially presented stimuli. By far the largest differences came from the sequentially presented stimuli. In that task, a number of matrices were presented one after the other with a single black square in each one, with participants having to recall where the squares were at the end. Given the need to remember a sequence of squares and this type of task’s similarity to some versions of the Corsi blocks, it would be very unsurprising if it placed reasonable demands on visual spatial-attention [44] and temporal sequencing that the static tasks did not. The results are thus likely to be compatible with others including Stefanac et al. [41], who found visual attention is more important than visual working memory capacity.

Finally, Nguyen et al. [45] examined the size of the brain’s V1 area, interhemispheric asymmetry, and the relationship between them and a visual search task designed to measure early sensory processing in this area. Weak differences between their dyslexic and control groups existed, while correlations using their entire sample between reading ability and both interhemispheric asymmetry and visual search efficiency were significant. However, they did not examine the dyslexic or control groups separately and visual inspection of their data suggests that the within group correlations would be close to zero, making the results and their specific relationship to the variables examined difficult to interpret.

## 5. Overall Summary

There are clearly a number of issues associated with visual attention and how it relates to reading (see the Supplementary Materials for more articles [46,47,48,49,50,51,52] that were reviewed and a short discussion on peripheral issues to those examined here). One is simply what task might actually capture the useful variance that is related to reading in the most meaningful manner. The task that clearly had the best predictive ability with the data and can be run quickly is the VAS task, although some arguments remain as to what it is measuring, and most of the new data we examined was from languages that are orthographically complex. Other tasks, such as visual search, attentional blink, and the IOR tasks, are clearly far better established in terms of what they measure given their usage over the past decades, but they were not such good predictors of reading performance. Differences between such tasks have also not always been exploited to make hypotheses about the systems used in reading, but rather just conglomerated together for more reliability. A larger review of these may find that differences in significance across studies may be of interest compared to the VAS task where differences are more ubiquitous. In this respect, if they consistently predicted performance on some groups but not others, one might potentially identify processes that children need to learn to read but which reach an asymptote. Similarly, if there are cross-language differences, something that requires more processing in one language compared to another could also be identified.

A second common issue was the lack of within-group correlations reported when control and dyslexic groups were compared. This meant that many issues could not be addressed. Many of the tasks also did not use RAN or PA as covariates, and there were only quite weak findings in Cirino et al. [20] when that was performed. Indeed, in none of the experiments we examined that did not use the VAS task were both of these tasks run. Some of the experiments we examined are thus open to the interpretation that differences found were due to covariation with other deficits and not the deficit actually examined.

Finally, from a practical point of view, it seems reasonable to suggest that if one wants to look at visual attention deficits in reading, running the VAS task is worthwhile. This is because many studies have found significant effects of it across quite disparate populations, it is quick to run, and the amount of variance that it captures that is unrelated to window size may be small. In addition, even if part of the variance it is capturing is related to phonological processing, a reasonable phonological task would presumably be able to control for this unless that part is truly unique. Finding null effects with it would also be informative [27]. In terms of tasks that can be used to measure other visual deficits and how they relate to different aspects of reading, the choices are less clear. From a theoretical point of view, there are still many open ends, not least of which is what some of the visual tasks are actually measuring and how differences between them might be used to test models of reading or find children with deficits in reading. Thus, the problem of what visual deficits are and how they manifest in the different processes that underlie reading is still yet to be understood as well as it could be.

## Supplementary Materials

The following supporting information can be downloaded at: https://www.mdpi.com/article/10.3390/brainsci12010087/s1. These materials include a short description and evaluation of studies to do with (a) ocular differences between dyslexic and normal readers; and (b) remediation of dyslexia and technology.
