# Supplementary material for: What Is Going on with Visual Attention in Reading and Dyslexia? A Critical Review of Recent Studies
Source: Brain Sci. 2022 Jan 10;12(1):87. doi: 10.3390/brainsci12010087 (PMC8773944; doi:10.3390/brainsci12010087)
Supplement: Supplementary file 1 [file brainsci-12-00087-s001.zip › brainsci-1529138-supplementary.pdf]

Supplementary Materials

Listing S1. Description and evaluation of additional studies

Data examining ocular movements or eye physiology

Both *Jiménez et al*. [46] and *Franzen et al*. [47] examined the oculomotor patterns of poor readers and children who read normally. They found a plethora of differences between them. Jiménez et al. came to the conclusion that the differences were likely to be cognitive (attention) and Franzen et al. suggested they were likely to reflect cognitive and physiological differences.

Schiavi et al. [48] examined M-Y ganglion cells of 10 adult dyslexics using a steady-state electroretinogram and frequency doubling perimetry and compared them to non-dyslexic controls. These cells are an input into the magnocellular system, a pathway in the brain thought to be very important in reading [1]. They found significant differences between dyslexics and their control group, with the dyslexics showing a lower amplitude as well as a difference between right and left eyes. They suggested that these differences would cause differences in visual attention usage between the groups.

Training studies may also provide insight into visual attention and ocular movements. In this respect, *Caldani et al.* [49] examined the reading performance of 50 French dyslexics (7.8-12 years old) given an ocular training program that lasted only 10 minutes. The results showed that it significantly sped up their reading times (around 20%). Such a dramatic decrease in such a small time may suggest that aberrant ocular processing may be caused by the use of inefficient motor patterns even though more reasonable ones already exist that could be used. This suggests that for some dyslexics, the problem may not be that they cannot learn normal patterns, but rather that they tend to use inefficient ones unless taught otherwise.

Training studies and technology

Bertoni et al. [50] examined 14 Italian children with developmental dyslexia (mean age: 8.93 years old) and the effect 12 hours of active video gaming had on text reading, pseudoword reading, phonological decoding, visual search, and non-alphanumeric RAN tasks. They found that training improved their performance on pseudowords, increased

phonological decoding ability and visual search speed. When a non-active gaming control was used, no significant benefits were found.

Zoubrinetsky et al. [51] examined the effect of trying to remediate the categorical perception of phonemes as well as VAS using two different computer packages (RapDys©, MAEVA©) with French children (mean age 10 years, 7 months). The first of these packages is meant to train phonemic discrimination and the second VAS. These were administered for 15 minutes a day across 6 weeks, and the children used them 5 days a week – 450 minutes of total training. The results showed both packages improved phonemic awareness and regular word reading, the RapDys package improved phonemic discrimination and pseudoword reading, and the Maeva package improved VAS and irregular word reading. Notably, Maeva caused VAS scores to go up by about 10% and RapDys cause phonemic discrimination scores to go up by about .7 using a d' measure. These results suggest that training packages for specific purposes tend to increase scores on aspects of reading they were supposed to.

Werth et al. [52] did not look at VAS specifically, but they used a computer program that broke words down into the number of letters German children (mean age 10 years and 2.4 months, 8-16 years old) could recognize at the same time. This was done by calibrating the number of letters each child could read from a task that presented pseudowords of different lengths. When they then read two different texts, one where spaces were placed between words and one where they were not, and their error rate dropped from 7.67% to 2.33%. Given the task, the extent to which it overlaps the VAS task would be interesting since it calculates the optimum sized letter windows people can use.

Overall, it appears that it is possible to train children on certain aspects of reading, and that these aspects may be relatively specific depending on the training program. However, correlations with actual reading scores and the effect of changes were not typically given, and, in the active video gaming studies, presumably many cognitive and perceptual process are affected. This makes more general theoretical conclusions from these studies hard to draw. To establish causation for more theoretically motivated questions, rather than use a non-active control for computer-game type remediation, a better control condition would be a similarly matched active control that is not predicted to help. For example, when computer games are used to increase visual attention abilities, one can imagine two versions: one where visual attention is important, and one which uses active gaming but where it is not as important. This would then control for whether simply playing active games causes an effect or whether it is really an effect of visual attention training.
